# Aquaglyceroporins: Drug Targets for Metabolic Diseases?

**DOI:** 10.3389/fphys.2018.00851

**Published:** 2018-07-10

**Authors:** Giuseppe Calamita, Jason Perret, Christine Delporte

**Affiliations:** ^1^Department of Biosciences, Biotechnologies and Biopharmaceutics, University of Bari Aldo Moro, Bari, Italy; ^2^Laboratory of Pathophysiological and Nutritional Biochemistry, Université Libre de Bruxelles, Brussels, Belgium

**Keywords:** adipose tissue, diabetes, drug, endocrine pancreas, liver, metabolic diseases, non-alcoholic fatty liver disease, obesity

## Abstract

Aquaporins (AQPs) are a family of transmembrane channel proteins facilitating the transport of water, small solutes, and gasses across biological membranes. AQPs are expressed in all tissues and ensure multiple roles under normal and pathophysiological conditions. Aquaglyceroporins are a subfamily of AQPs permeable to glycerol in addition to water and participate thereby to energy metabolism. This review focalizes on the present knowledge of the expression, regulation and physiological roles of AQPs in adipose tissue, liver and endocrine pancreas, that are involved in energy metabolism. In addition, the review aims at summarizing the involvement of AQPs in metabolic disorders, such as obesity, diabetes and liver diseases. Finally, challenges and recent advances related to pharmacological modulation of AQPs expression and function to control and treat metabolic diseases are discussed.

## Introduction

Mammalian aquaporins (AQPs) are a family of 13 integral membrane channel proteins termed AQP0–AQP12, facilitating the transport of water, small solutes, and gasses across biological membranes (Agre, 2004; Verkman, 2005). Comparatively, transport properties among the different AQPs are quite heterogeneous, reason why their exhaustive classification represents a difficult task. Mammalian AQPs are roughly subdivided in three subfamilies: (1) the classical AQPs, permeable to water (AQP0, AQP1, AQP2, AQP4, AQP5, AQP6, and AQP8) (Agre, 2004; Verkman, 2005); (2) the aquaglyceroporins, permeable to small solutes including urea and glycerol, in addition to water (AQP3, AQP7, AQP9, and AQP10) (Agre, 2004; Verkman, 2005; Rojek et al., 2008); and (3) unorthodox AQPs with suggested permeability to water (AQP11, AQP12) and glycerol (AQP11) (Ishibashi et al., 2014). Some AQPs are also permeable to H_2_O_2_ (AQP1, AQP3, AQP5, AQP8, and AQP9) and/or ammonia (AQP1, AQP3, AQP4, AQP6, AQP7, AQP8, and AQP9) (Jahn et al., 2004; Geyer et al., 2013), and for these biophysical properties they are also called peroxiporins (Almasalmeh et al., 2014) and ammoniaporins (or aquaammoniaporins) (Holm et al., 2005; Saparov et al., 2007; Almasalmeh et al., 2014), respectively. Among aquaglyceroporins, AQP9 exhibit broad selectivity toward several neutral solutes including urea, carbamides, nucleosides, monocarboxylates, pyrimidines, purines and metalloid arsenic in addition to glycerol and other polyols (Tsukaguchi et al., 1998; Liu et al., 2002).

AQPs also allow movement of gasses such as CO_2_ (AQP1, AQP4, and AQP5), NO (AQP1, AQP4) or O_2_ (AQP1, AQP4) (Herrera et al., 2006; Saparov et al., 2007; Wang et al., 2007; Boron, 2010).

The expression of aquaglyceroporins has been documented in many body locations including adipose tissue, liver, and pancreas where they are reported to play important metabolic functions (Rojek et al., 2008). This review aims to present the current knowledge on the role of aquaglyceroporins in adipose tissue, liver and endocrine pancreas, and their possible use as drug targets for metabolic diseases.

## Role of Aquaglyceroporins in Adipose Tissue

### Physiological Conditions

Considering the role of aquaglyceroporins in adipose tissue has been subjected to extensive reviews (see Frühbeck et al., 2006; Maeda et al., 2009; Madeira et al., 2015; Laforenza et al., 2016; da Silva and Soveral, 2017), a brief summary is given here after on this topic. The first adipose aquaglyceroporin that has been identified in human, mouse and rat is AQP7 (Ishibashi et al., 1997, 1998; Kishida et al., 2001; Kondo et al., 2002). Differentiation of mice 3T3-L1 cells into adipocytes leads to an increased expression in *Aqp7* mRNA (Arsenijevic et al., 2013; Miyauchi et al., 2015; Chiadak et al., 2016) and AQP7 protein (Chiadak et al., 2017). Differentiation of human preadipocytes into adipocytes also leads to an increased expression in both *Aqp7* mRNA and protein (Miranda et al., 2010). While studies reported AQP7 expression exclusively in endothelial cells surrounding adipocytes (Skowronski et al., 2007; Lebeck et al., 2012b), other studies reported AQP7 expression in both endothelial cells and adipocytes (Laforenza et al., 2013; Miyauchi et al., 2015). Subsequently, other aquaglyceroporins have been detected in human adipocytes: AQP3 (Rodríguez et al., 2011), AQP9 (Rodríguez et al., 2011), and AQP10 (Laforenza et al., 2013). However, the presence of AQP3, AQP9, and AQP10 in human adipose tissue was not supported by all studies as Miranda et al. (2010) only detected AQP7 and Lindskog et al. (2016) failed to detect AQP9. In mouse 3T3L-1 cells differentiated into adipocytes, the presence of AQP3, AQP7, and AQP9 has also been found (Chiadak et al., 2016, 2017). In mouse 3T3-L1 cells differentiated into adipocytes, no modification in *Aqp3* and *Aqp9* mRNA levels were detected (Chiadak et al., 2016), while both AQP3 and AQP9 protein levels were increased (Chiadak et al., 2017). On the other hand, some studies did not show the presence of AQP3 and AQP9 in mouse adipose tissue (Kishida et al., 2000; Maeda et al., 2004) or 3T3-L1 cells differentiated into adipocytes (Kishida et al., 2000). In addition, AQP11, an unorthodox AQP, has recently been shown to be permeable to glycerol and present in human and mouse adipocytes (Madeira et al., 2014b; Chiadak et al., 2016). While AQP10 is expressed in human, it is a nonfunctional pseudogene in mice (Morinaga et al., 2002). In human adipocytes, AQP10, localized in the cytoplasm and the lipid droplets, undergoes translocation to the plasma membrane upon isoproterenol stimulation (inducing lipolysis), suggesting AQP10 could be involved in glycerol exit during lipolysis (Laforenza et al., 2013). In rat adipocytes, AQP3 expression has been detected while, to our knowledge, expression of AQP9 and AQP10 expression have not yet been reported (Méndez-Giménez et al., 2014).

In adipocytes, aquaglyceroporins display different subcellular localizations and are subjected to trafficking in response to hormonal stimulation, including insulin and cAMP-inducing hormones (Rodríguez et al., 2011, 2015a; Miyauchi et al., 2015; Hansen et al., 2017). Aquaglyceroporins present in adipose tissue are transcriptionally regulated by hormones (such as insulin, catecholamines, and steroids) and inflammatory mediators (such as TNFα, and lipopolyssacharides) (Kishida et al., 2001; Kondo et al., 2002; Fasshauer et al., 2003; Rodríguez et al., 2011; Chiadak et al., 2016). In addition, human *Aqp7* and mouse *Aqp7* transcriptional activity is upregulated by peroxisome proliferator-activated receptor gamma (PPARγ) (Kishida et al., 2001; Lindgren et al., 2002). Finally, a very recent study showed that *Aqp7* transcription is also controlled by the transcription factor Küppel-like factor 15 (KLF-15), known be involved in insulin-triggered lipogenesis (Kulyté et al., 2017).

From a metabolic point of view, adipose tissue is a major source of triacylglycerols (TAG) storage during feeding, as well as a major source of plasma glycerol during fasting (Reshef et al., 2003; Arner, 2005). Indeed, both TAG synthesis and storage are promoted during feeding, while TAG degradation occurring during lipolysis upon TAG lipase activation is promoted during fasting.

Experiments using differentiated adipocytes (Kishida et al., 2000) and *Aqp7* knockout mice (Maeda et al., 2004; Hara-Chikuma et al., 2005; Hibuse et al., 2005) evidenced the role of AQP7 in glycerol release from adipocytes. AQP3 and AQP9 were also proposed to play a role in glycerol release during lipolysis or in glycerol intake during lipogenesis, respectively (Rodríguez et al., 2011). Following isolation from omental adipose tissue, human stromovascular fraction cells expressed approximately a 12-fold higher *AQP3* mRNA level as compared to adipocytes, suggesting that AQP3 is predominantly expressed by other cell types (e.g., preadipocytes, macrophages, fibroblasts, erythrocytes, neutrophils, and leukocytes) than in adipocytes from adipose tissue (Rodríguez et al., 2011). The involvement of AQP9 in adipocyte glycerol uptake has been suggested by several data. Firstly, the expression of AQP9, localized at the plasma membrane from mouse 3T3-L1 cells differentiated into adipocytes, and increased in response to insulin (Rodríguez et al., 2011). Secondly, in human omental adipocytes, the expression of AQP9, but also of AQP3 and AQP7, is increased by insulin, while the expression of AQP9, and also of AQP7, is decreased by leptin though the phosphatidylinositol 3-kinase (PI3K)/protein kinase B (Akt)/mammalian target of rapamycin (mTor) pathway (Rodríguez et al., 2011). However, during lipogenesis, glycerol uptake is likely limited due to the low activity of the adipose glycerol kinase (GK).

In mouse 3T3-L1 cells differentiated into adipocytes, hormones activating the cAMP pathway induced the translocation of AQP3 and AQP7, localized within intracellular vesicles, to the plasma membrane, while the plasma membrane localization of AQP9 was unchanged (Rodríguez et al., 2011). Insulin induced an increased intracellular localization of AQP3 and AQP7 and an increased plasma membrane localization of AQP9 in 3T3-L1 cells differentiated into adipocytes (Rodríguez et al., 2011) (as stated above). Using selective AQPs inhibitors, it was possible to estimate biophysically the relative contributions of AQP3, AQP7, and AQP9 to the glycerol permeability (measured as *Ki*, an index reflecting the membrane glycerol permeability) of plasma membrane vesicles prepared from mouse 3T3-L1 cells differentiated into adipocytes were estimated to 68, 3, and 12%, respectively (Chiadak et al., 2017). The relative contributions of AQP3, AQP7, and AQP9 to the membrane glycerol permeability were estimated using the following formulas; [(*Ki* in the absence of inhibitor – *Ki* in the presence of CuSO_4_)/(*Ki* in the absence of inhibitor)]; [(*Ki* in the presence of HgCl_2_ – *Ki* in the presence of phloretin)/(*Ki* in the absence of inhibitor)]; [*Ki* measured in the presence of CuSO_4_ – *Ki* in the presence of HgCl_2_)/(*Ki* in the absence of inhibitor)]. In 3T3-L1 cells differentiated into adipocytes, the low (3%) relative contribution of AQP7 to glycerol permeability (Chiadak et al., 2017) might be underestimated due to possible incomplete effects of the inhibitors, while the important (45%) reduction in glycerol release in the cell media following AQP7 knockdown using RNAi might be overestimated due to possible off target effects of RNAi (Hibuse et al., 2005). Based on these data, an updated model of the role of aquaglyceroporins in the regulation of lipogenesis and lipolysis was recently proposed: whereby mainly AQP3 and AQP7 would play a role in glycerol release during lipolysis, while mainly AQP9 would play a role in limited glycerol uptake that may occur during lipogenesis (Chiadak et al., 2017). Understanding the role of each aquaglyceroporin in adipose tissue remains to be fully elucidated using mice made deficient, in a tissue-specific manner, for one or multiple aquaglyceroporin gene(s).

**FIGURE 1:**
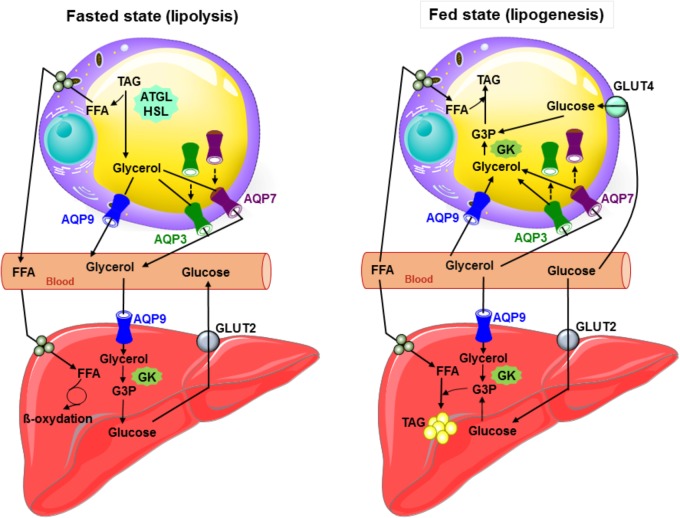
Glycerol metabolism: working model of the interplay between the liver and adipose tissue in fed and starved states in mouse. During fasted state, glucagon secretion is induced, TAG are hydrolyzed to glycerol and free fatty acids in adipocytes, and both AQP7 and AQP3 traffic to the plasma membrane and participate with AQP9 to glycerol efflux. AQP9 allows plasma glycerol entry in hepatocytes. Glycerol is then converted to glycerol-3-phosphate by glycerol kinase, and thereby participates to gluconeogenesis. During fed state inducing insulin secretion, glucose metabolites, glycerol-3-phosphate (obtained from glycerol by glycerol kinase enzymatic activity) and free fatty acids are used to produce TAG in both liver and adipose tissue. In response to insulin in adipocytes, AQP3 and AQP7 traffic from the plasma membrane to intracellular compartments. Glycerol most likely mainly enters adipocytes via AQP9. During lipogenesis, adipose glycerol uptake is likely low due to the weak activity of the glycerol kinase. In hepatocytes, glycerol likely enters via AQP7 and AQP9. ATGL, adipose triglyceride lipase; GK, glycerol kinase; Glut2, glucose transporter 2; Glut4, glucose transporter 4; G3P, glycerol-3-phosphate; FFA, free fatty acids; HSL, hormone sensitive lipase; TAG, triacylglycerols; broken arrows: trafficking.

**Figure 1** summarizes the roles played by aquaglyceroporins in adipose tissue under fasted and fed states in mouse.

### Metabolic Diseases

In adipose tissue, the role of aquaglyceroporins in metabolic diseases, and more especially in obesity and metabolic syndrome, has extensively been reviewed (Frühbeck et al., 2006; Maeda et al., 2009; Lebeck, 2014; Madeira et al., 2015; Laforenza et al., 2016; da Silva and Soveral, 2017). Therefore, only a brief overview will be presented on this topic.

Depending on their genetic background, *Aqp7* knockout mice were shown to either develop or not develop obesity (Hara-Chikuma et al., 2005; Hibuse et al., 2005; Matsumura et al., 2007). The site of AQP7 expression, exclusively in endothelial cells (Skowronski et al., 2007; Lebeck et al., 2012b) or in endothelial cells and adipocytes (Laforenza et al., 2013; Miyauchi et al., 2015), could account for the susceptibility of mice to develop obesity (Skowronski et al., 2016). In n3-polyunsaturated fatty acids (PUFA) depleted rats, an animal model of metabolic syndrome, a decrease in AQP7 protein levels was observed without any modification in *Aqp7* mRNA levels [possibly due to the lack of PPARγ activation concomitant to PUFA depletion, as PPARγ normally activates the transcription of AQP7 (Kishida et al., 2001)] and glycerol transport (Portois et al., 2012b).

As human *AQP7* gene is localized in a chromosomal region with reported linkage to type 2 diabetes (T2D) (Luo et al., 2001; Lindgren et al., 2002) and the metabolic syndrome (Loos et al., 2003), AQP7 expression has been particularly studied in patients suffering from obesity, T2D and metabolic syndrome. *AQP7* gene missense and silent mutations were not correlated with obesity and T2D (Kondo et al., 2002). In adipose tissue, deregulation of AQP7 expression has been documented in obese subjects, with studies presenting opposing results reporting either a decrease or increase in AQP7 expression (Marrades et al., 2006; Ceperuelo-Mallafré et al., 2007; Prudente et al., 2007; Catalán et al., 2008; Miranda et al., 2009; Rodríguez et al., 2011; de Luis et al., 2017). These apparent contradictory data might be related to the clinical characteristics of the obese phenotype of the obese subjects, as well as their gender (Rodríguez et al., 2015b). Very recently studies have shown that insulin resistance of adipose tissue could be related to lower KLF-15 expression that could reduce *AQP7* expression (Kulyté et al., 2017). On the other hand, lipopolysaccharide, known to play an important role in obesity (Boutagy et al., 2016), decreased glycerol permeability and increased TAG content in 3T3-L1 adipocytes, without modification of AQP7 protein expression (Chiadak et al., 2017). Species-related expression and proposed physiological and pathophysiological roles of adipose tissue aquaglyceroporins are summarized in **Table 1**.

**Table 1:** Reported species-related expression and suggested physiological and pathophysiological relevance of adipose tissue aquaglyceroporins.

Aqua-glyceroporin	Cell type	Suggested physiological role	Suggested pathophysiological involvement
AQP3	Adipocytes (h, m, r)	TAG synthesis; uptake of glycerol (low contribution)	
		Lipolysis; release of glycerol	
AQP7	Adipocytes (h, m, r)	TAG synthesis; uptake of glycerol (low contribution)	Obesity; Insulin resistance; metabolic syndrome
		Lipolysis; release of glycerol	
	Endothelial cells (h, m)	Unknown	
AQP9	Adipocytes (h, m)	TAG synthesis; uptake of glycerol (low contribution)	
		Lipolysis; release of glycerol	
AQP10	Adipocytes (h)	Unknown	

*h, human; m, mouse; r, rat.*

## Role of Aquaglyceroporins in Liver

### Physiological Conditions

#### Roles of Aquaglyceroporins in Liver Glycerol Metabolism and Metabolic Homeostasis

Glycerol is an intermediate metabolite of physiological relevance in energy metabolism. Indeed, glycerol is a direct source of glycerol-3-phosphate (G3P), an important substrate for hepatic gluconeogenesis during fasting and for TAG synthesis (Brisson et al., 2001; Reshef et al., 2003). Glycerol can derive from G3P (Mugabo et al., 2016), produced by glucose use through glycolysis, or the conversion of pyruvate, lactate and alanine through gluconeogenesis. Liver plays a central role in glycerol metabolism, due to its major (70–90%) contribution to the whole-body glycerol metabolism (Reshef et al., 2003). To different extents and with different cellular distributions, liver expresses all four aquaglyceroporins, with AQP9 being by far the most expressed.

In rodents and human, AQP9 is expressed at the sinusoidal domain of the hepatocyte plasma membrane, facing the space of Disse (Elkjaer et al., 2000; Carbrey et al., 2003; Lindskog et al., 2016). In human, AQP9 expression is higher in hepatocytes, as compared to the other few tissues in which it is found (Lindskog et al., 2016). In both rodents and human, AQP9 represents the major pathway for glycerol import from portal blood to hepatocytes (Jelen et al., 2011; Calamita et al., 2012). The membrane permeation represents the rate limiting step in glycerol use by the liver (Li and Lin, 1983). Once into the hepatocyte, glycerol is promptly converted into G3P by GK. In short term fasting, G3P is particularly important as a substrate for *de novo* synthesis of glucose (gluconeogenesis) (Brisson et al., 2001; Jelen et al., 2011; Calamita et al., 2012; Calamita et al., 2015; Bernardino et al., 2016). In rodents, liver *Aqp9* is transcriptionally downregulated by insulin (Kuriyama et al., 2002), explaining why streptozotocin-induced type 1 diabetes in rodents (Carbrey et al., 2003) and insulin resistance in human (Rodríguez et al., 2014) are associated with an increase in the hepatic levels of AQP9. Consistently, ablation of *Aqp9* in obese diabetic leptin receptor-deficient *db*/*db* mice decreases plasma glucose levels by 10–40% (Rojek et al., 2007). A model for the hepatic glucose metabolism based on Hill and step functions was recently devised as a way to integrate the hepatic entry and handling of glycerol in the glucose-insulin axis taking into account the (i) refilling/depletion of glycogen stores during the feeding or fasted state, (ii) the influence of plasma glycerol and (iii) the liver glycerol permeability to hepatic AQP9 protein levels (D’Abbicco et al., 2016). To follow, a system of first-order ordinary differential equations was devised delineating the dynamical involvement of AQP9 in mouse liver glycerol permeability (Gena et al., 2017). Thus, assuming the liver glycerol permeability depends on the levels of AQP9 in hepatocyte, a mathematical function was generated describing the time course with which AQP9 is involved in mouse hepatic glycerol metabolism under different nutritional conditions. The theoretical relationship was derived fitting experimental data obtained with mice in the fed, starved or re-fed states. Such a model, appropriately adapted to the human liver, has good potentials to be used as a whole body-model for glucose metabolism in normal and metabolic disorders.

Liver AQP9 has also considerable relevance to lipid metabolism as G3P also represents a key substrate for TAG synthesis (Rodríguez et al., 2011; Lebeck, 2014). Coordinately, AQP9-depleted (*Aqp9*^−/−^) mice display reduced liver glycerol permeability and higher levels of plasma glycerol and TAG compared to wild type (WT) (*Aqp9*^+/+^) mice (Rojek et al., 2007; Calamita et al., 2012). Liver AQP9 is also controlled transcriptionally by leptin (Rodríguez et al., 2011, 2015c), however, surprisingly, the regulations exerted by both insulin and leptin on AQP9 appear to be distinct when comparing rodents with humans (Lebeck, 2014; Calamita et al., 2015). In HepG2 cells, a human hepatocellular carcinoma cell line, insulin increased while leptin decreased AQP9 expression through the activation of the phosphatidylinositol 3-kinase/protein kinase B/mammalian target of rapamycin (PI3K/Akt/mTOR) signaling cascade (Rodríguez et al., 2011) and AMP protein kinase (AMPK), via forkhead box a2 (Fox a2) (Yokoyama et al., 2011). However, the regulation exerted by both insulin and leptin on human AQP9 expression remains an open question considering that a recent work did not detect AQP9 expression in HepG2 cells (Lindskog et al., 2016). Additional work is therefore needed to fully assess the way by which insulin and leptin modulate AQP9 expression in human liver.

In rat, the fasting-induced increase of liver AQP9 expression resulted a 2.6 times higher level in males than females (Lebeck et al., 2012a). Consistently, during fasting, male rats displayed unmodified plasma glycerol levels, whereas female rats displayed increased plasma glycerol levels. This was paralleled by the higher liver glycerol permeability in males than in females. Ovariectomy led to a fasted-induced profile comparable to that observed in male rats with increased liver AQP9 expression and unmodified plasma glycerol levels. These observations, together with the data acquired with the studies using cultured hepatocytes exposed to 17β-estradiol and an estrogen receptor β-agonist, suggest that the gender specific regulation of AQP9 during fasting contributes to the higher levels of plasma glycerol found in female rats than in male rats (Lebeck et al., 2012a). Decreased AQP9 protein levels were found in periportal hepatocytes of male rats in response to the peroxisome proliferator-activated receptor α (PPARα) (Lebeck et al., 2015). Obese women showed lower liver glycerol permeability compared to obese men whereas the hepatic levels of AQP9 between the two genders were comparable (Rodríguez et al., 2014). This significant observation may help explain why insulin resistance and Non-Alcoholic Fatty Liver Disease (NAFLD) have lower incidence in women than men. Gender-specific differences have been found as well for AQP3 and AQP7, two other aquaglyceroporins, with metabolic relevance in the adipose tissue (for review see Rodríguez et al., 2015b).

Probably due to the broad selectively of its channel, hepatocyte AQP9 seems to be of pleiotropic relevance, as roles in bile formation (Calamita et al., 2008; Portincasa and Calamita, 2012) and hepatic extrusion of catabolic urea (Jelen et al., 2012) have been reported. AQP9 has also been described to allow excretion of arsenic from the liver (Carbrey et al., 2009). AQP9 immunoreactivity has been also detected in the cholangiocytes lining the intrahepatic bile ducts (Gregoire et al., 2015), however, the related physiological significance remains undefined.

In addition to AQP9, but to a lesser extent and undermined by conflicting results, human hepatocytes have been also reported to express the other three aquaglyceroporins, AQP3, AQP7, and AQP10 (Rodríguez et al., 2014; Gregoire et al., 2015). Women showed increased hepatic transcript levels of *AQP3* compared to men, but no gender dimorphism in the liver gene expression of *AQP7* and *AQP10* was observed (Rodríguez et al., 2014). Leptin upregulated AQP3 and downregulated AQP7 in the HepG2 hepatocytes (Rodríguez et al., 2011). However, in spite of these observations, the physiological meaning of hepatocyte AQP3, AQP7, and AQP10 in humans, if any, remains elusive. Human hepatic stellate cells (HSC) express AQP3 whose levels were reported to decrease during cell activation (Tardelli et al., 2017a,b). AQP3 is also found in human Kupffer cells (Gregoire et al., 2015) where it may play a role in cell repopulation during liver regeneration and be involved in the migration and proinflammatory secretion of cytokines that these cells undergo in the course of liver pathologies. Overall, the cellular and subcellular localization of AQP3, AQP7, and AQP10 in liver does not seem to overlap with that of AQP9, and additional studies are warranted to fully clarify their roles in liver. AQP9 (Tietz et al., 2005), in addition to AQP1 (Marrone et al., 2016), and AQP8 (Tietz et al., 2005; Marrone et al., 2016), has been located in rat hepatocyte membrane microdomains enriched in high-cholesterol, sphingomyelin and caveolin-1. Upon glucagon stimulation, the presence of AQP8, but not of AQP9, was increased in these membrane microdomains (Tietz et al., 2005).

The reported localization and suggested physiological relevance of liver aquaglyceroporins are described in **Table 2**. **Figure 1** summarizes the roles played by AQP9 in liver under fasted and fed states in mouse.

**Table 2:** Reported localization and proposed physiological and pathophysiological relevance of liver aquaglyceroporins.

Aquaglyceroporins	Cell type	Subcellular location	Suggested physiological role	Suggested pathophysiological involvement
AQP3	Kupffer cells (h)	Undefined	Repopulation of Kupffer cells in liver regeneration	Kupffer cell migration and proinflammatory cytokine secretion
	Hepatocytes (h)	Undefined	Uptake of glycerol?	Insulin resistance?
	Stellate cells (h)	Undefined	Cell activation	Hepatic steatosis (I148M variant)
AQP7	Hepatocytes (h, m)	Cyt; others?	TAG synthesis; uptake of glycerol?	Insulin resistance
	Cholangiocytes (h)	APM	Unknown	
	Endothelial cells (h)	APM	Unknown	
AQP9	Hepatocytes (h, m, r)	BLPM	Uptake of portal glycerol (gluconeogenesis; TAG synthesis); import of water from portal blood (bile formation); urea extrusion; arsenic detoxification; liver regeneration	NAFLD; NASH; obesity; T2D; hepatocellular carcinoma
	Cholangiocytes (h)	Undefined	Unknown	
AQP10	Hepatocytes (h)	Undefined	Exit of glycerol during lipolysis	

*APM, apical plasma membrane; BLPM, basolateral plasma membrane; h, human; m, mouse; NAFLD, non-alcoholic fatty liver disease; NASH, non-alcoholic steatohepatitis; r, rat; TAG, triacylglycerols; T2D, type 2 diabetes mellitus.*

### Liver Aquaglyceroporins in Disease

Consistent with their role in facilitating glycerol transport into and out of cells and the related control of fat accumulation and glucose homeostasis, hepatic aquaglyceroporins, in particular AQP9, are reported to be implicated in metabolic disorders affecting the liver (**Table 2**). The roles in metabolic diseases played by the hepatic AQPs belonging to the classic and unorthodox groups of AQPs have been reviewed elsewhere (Portincasa et al., 2008).

#### Liver Aquaglyceroporins in Fatty Liver Disease, Obesity, Diabetes Mellitus and Hepatocellular Carcinoma

The pathology of NAFLD is multifactorial and characterized by ectopic accumulation of TAG in the liver ranging from simple fatty liver (hepatic steatosis) to NASH and to cirrhosis (irreversible, advanced scarring of the liver) (Chalasani et al., 2012). NAFLD is often associated with the metabolic syndrome often characterized by obesity and diabetes mellitus with insulin resistance. NAFLD and NASH are the object of intensive investigation, especially regarding the pathogenic pathways leading to excessive TAG accumulation within the liver parenchyma (Tiniakos et al., 2010). AQP9 is hypothesized to be involved in the hepatic synthesis of TAG in NAFLD as indicated by (i) the amelioration of high fat diet-induced NAFLD in rats with knockdown of hepatic AQP9 (Cai et al., 2013), (ii) the increase in hepatocyte AQP9 expression accompanying the development of fatty liver in diet-induced obese (DIO) mice (Hirako et al., 2016), (iii) the impairment of hepatocyte AQP9 and glycerol permeability found in an animal model of NAFLD [leptin-deficient mice (*ob*/*ob* mice)] (Gena et al., 2013) and in patients with obesity, insulin-resistance and NAFLD (Rodríguez et al., 2014). However, some data do not support this hypothesis as *db/db* mice made AQP9 knockdown had similar TAG content as *db/db* mice (Spegel et al., 2015), and as unmodified AQP9 expression was found in obese *ob/ob* mice (Rodríguez et al., 2015c). The downregulation of AQP9 during obesity and/or diabetes could represent a compensatory mechanism selected at decreasing substrate availability for *de novo* TAG synthesis, thereby counteracting the ectopic accumulation of TAG into hepatocytes, and reducing gluconeogenesis, thereby thwarting the progression of hyperglycemia (Gena et al., 2013; Rodríguez et al., 2014, 2015b). However, in patients with morbid obesity, no relationship could be found between AQP9 expression and the degree of hepatic steatosis or fibrosis (Miranda et al., 2009). N3-PUFA (ω3 polyunsaturated fatty acids)-depleted female rats (an animal model of metabolic syndrome displaying various features of the disease including the hepatic over-accumulation of TAG) showed reduced AQP9 protein levels and increased hepatic glycerol uptake as compared to control animals (Portois et al., 2012a). It is therefore conceivable that AQP9 increases early during onset of steatosis while decreases at a later stage of the disease, when fat accumulation becomes excessive. Thus, the involvement of AQP9 in fatty liver should be contextualized within the origin of the disease, pathogenic profile and sex of the investigated animals and subjects. Further studies are therefore required to fully assess the etiopathological involvement and modulation of AQP9 in NAFLD-NASH. Nevertheless, the pharmacological interest toward liver AQP9 is strong as this aquaglyceroporin has good potentials to be a novel molecular target for therapeutic intervention in NAFLD and NASH. While selective small molecule AQP9 inhibitors with low micromolar IC50 values have already been reported and work is ongoing to increase the aqueous solubility of such blockers (Jelen et al., 2011; Wacker et al., 2013), preliminary studies using NAFLD cell models showed that silencing of AQP9 prevents or alleviates the degree of steatosis (Wang et al., 2013).

AQP9 seems to be of minor pathophysiological relevance in the fatty liver disease of alcoholic origin. A rapid increase in glycerol import and cell size was seen when rat primary hepatocytes were acutely exposed to acetaldehyde, an oxidation product derived from ethanol by the action of the hepatic enzyme alcohol dehydrogenase (Potter et al., 2011). The acute effects of acetaldehyde, while mediated by AQP9, were interpreted as mostly due to the binding of acetaldehyde to hepatocyte membranes and consequent changes in cell permeability. Exposure to ethanol, in spite of not changing AQP9 expression, increased the activity of GK and PEPCK (phosphoenolpyruvate carboxykinase), leading to increased production of G3P, which thereby could contribute to alcoholic hepatic steatosis (Potter et al., 2011).

Contrary to liver steatosis, a recent work using subcutaneously xenografted liver tumors in nude mice showed that hepatic AQP9 overexpression inhibit hepatocellular carcinoma by upregulating forkhead-box protein O1 (FOXO1) expression (Li et al., 2016). Decreased AQP9 expression was shown in hepatocellular carcinoma (Zhang et al., 2016). Novel strategies based on clinically feasible approaches may be developed to restore AQP9 expression for the prevention and treatment of hepatocellular carcinoma.

A recent study showed that treatment with estrogen protects against ovariectomy-induced hepatic steatosis by increasing hepatocyte AQP7 expression (Fu et al., 2016). Liver AQP7 was therefore suggested to play an important role in lipogenesis representing a potential target for the prevention and treatment of fatty liver disease in postmenopausal women.

Human HSC AQP3 was suggested to interact with the PNPLA3 I148M variant in causing hepatic steatosis, NASH, fibrosis and cancer (Tardelli et al., 2017a). A profound reduction of AQP3 in HSC carrying the PNPLA3 I148M polymorphism correlated with decreased PPARγ activation, which could be rescued by rosiglitazone, a PPARγ agonist, and blocking of JNK. This finding triggers the appealing idea that targeting AQP3 within HSCs activation may lead to the development of new treatments for liver fibrosis in PNPLA3 I148M patients.

## Role of Aquaglyceroporins in Endocrine Pancreas

### Physiological Conditions

Endocrine pancreas accounts for 10% of total pancreas and is made of islets of Langerhans dispersed within the pancreatic tissue. Islets of Langerhans are constituted of five types of cells: (1) β-cells producing insulin; (2) α-cells producing glucagon; (3) δ-cells producing somatostatin; (4) γ-cells producing pancreatic polypeptide; and (5) 𝜖-cells producing ghrelin (Langerhans, 1869; Andralojc et al., 2009; Jain and Lammert, 2009). Endocrine pancreas participates to the control of fuel metabolism by secreting insulin in post-prandial state, and glucagon in pre-prandial state.

To the best of our knowledge, the expression of aquaglyceroporins in human endocrine pancreas has not yet been documented. However, AQP7 is expressed in rat and mouse β-cells (Matsumura et al., 2007; Best et al., 2009; Louchami et al., 2012). While *Aqp3* and *Aqp9* mRNA were undetectable in mouse β-cells (Matsumura et al., 2007), this was also the case in rats with the exception of *Aqp9* mRNA that was detectable in 10% of the studied samples (Méndez-Giménez et al., 2017). Additional studies are necessary to clearly assess the presence of other aquaglyceroporins besides AQP7 in β-cells.

In β-cells, AQP7 has been shown to play a role in intracellular glycerol content and both insulin production and secretion (**Table 3**). As compared to WT mice, *Aqp7* knockout mice display a reduction in β-cells number, size and insulin content (Matsumura et al., 2007). In addition the *Aqp7* knockout mice presented increased intraislet concentration of glycerol and TAG, as well as increased GK activity, but abolished forskolin-induced glycerol release (Matsumura et al., 2007).

**Table 3:** Reported species-related expression and suggested physiological and pathophysiological relevance of pancreas aquaglyceroporins.

Aqua-glyceroporin	Cell type	Suggested physiological role	Suggested pathophysiological involvement
AQP3	Absent in β-cells (m, r)		
AQP7	β-cells (m, r)	Insulin secretion	Obesity
		TAG synthesis; uptake of glycerol	
		Lipolysis; release of glycerol	
		Regulation of β-cell size and number	
AQP9	β-cells (r)^∗^		
AQP10	Undefined		

*m, mouse; r, rat; ^∗^present in ± 10% of samples.*

Glycaemia was measured in several *Aqp7* knockout mice models. However, depending on their genetic background, these *Aqp7* deficient mice presented different phenotypes characterized by either normal glycaemia with underdetermined insulinemia (Skowronski et al., 2007), normal glycaemia with hyperinsulinemia (Matsumura et al., 2007), or hyperglycaemia and hyperinsulinemia (Hibuse et al., 2005). One can hypothesize that the resulting phenotype of *Aqp7* knockout mice results from combined interactions between *Aqp7* and various other gene products; unraveling the *Aqp7* interactome, therefore will be necessary for a better understanding.

**FIGURE 2:**
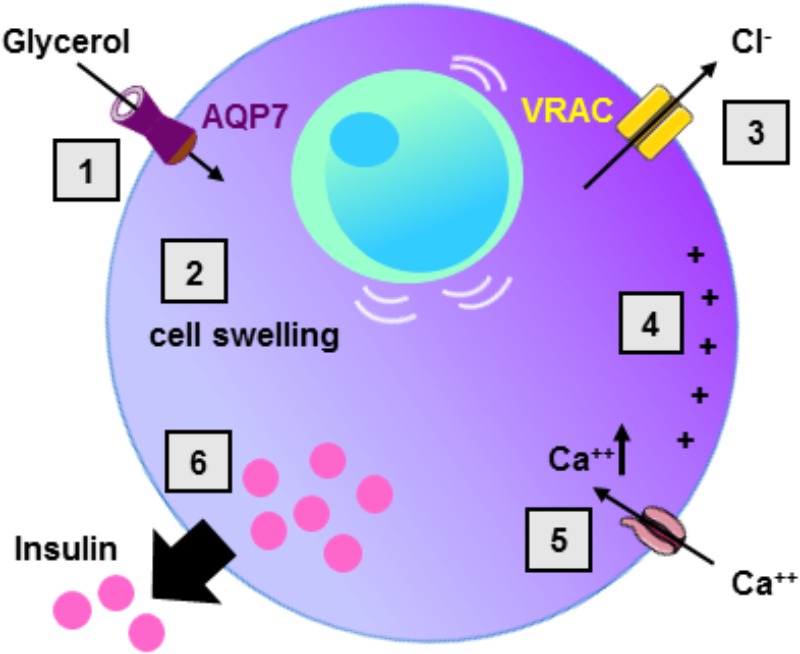
Role of AQP7 in β-cell insulin secretion. Following glycerol entry via AQP7 in β-cell (1), β-cell undergo cell swelling (2) leading to the activation of volume-regulated anion channel (VRAC) with concomitant Cl^-^ exit (3) and subsequent cell membrane depolarization (4). Cell membrane depolarization then activates voltage-sensitive calcium channels leading to intracellular calcium increase (5) provoking insulin exocytosis (6).

In the current model of insulin secretion, the sequential mechanisms accounting for insulin secretion involve: glucose uptake ensured by the glucose transporter type 2 (GLUT2), metabolism of glucose, increase in intracellular ATP concentration, inhibition of ATP-sensitive potassium channels, membrane depolarization, opening of voltage-dependent calcium channels, increase in intracellular calcium concentration, and finally insulin vesicle degranulation (Henquin, 2009) (**Figure 2**). During the glucose-induced insulin secretion, glucose also induces β-cell swelling that may affect cell activity (Miley et al., 1997). Indeed, in response to extracellular hypotonicity, β-cells swelling induces the activation of volume-regulated anion channels (VRAC), leading to subsequent cell depolarization, activation of voltage-dependent calcium channel, increase in intracellular calcium concentration, and finally insulin release (Best et al., 1996; Drews et al., 2010). When β-cells or BRIN-DB 11 cells are exposed to extracellular isosmotic addition of glycerol, cells undergo cell swelling, VRAC activation, plasma membrane depolarization, electrical activity and insulin release (Best et al., 2009; Delporte et al., 2009; Virreira et al., 2011). This activation of β-cell is likely due to both glycerol entry and metabolism (Best et al., 2009). Exposure of β-cells from *Aqp7* knockout mice, to either extracellular isosmotic addition of glycerol, extracellular hypotonicity or increased D-glucose concentration causes decreased insulin release as compared β-cells from WT mice (Louchami et al., 2012). The involvement of glycerol and AQP7 in the regulation of insulin secretion is in agreement with previous studies showing that glucose-stimulated insulin secretion correlates with β-cell lipolysis (Yaney and Corkey, 2003; Mulder et al., 2004; Winzell et al., 2006). Therefore, it is hypothesized that AQP7 plays a dual role in the regulation of insulin secretion by participating to glycerol flow (entry and exit), and by directly or indirectly acting at a distal downstream site in the insulin secretion pathway (Louchami et al., 2012) (**Table 3**).

Based on the results from the literature, following an increase in circulating glycerol levels (in response to the induction of lipolysis in adipocytes), it is hypothesized that local β-cells intracellular glycerol concentration rises and induces activation of β-cells. In addition, β-cells activation, subsequent to intracellular glycerol concentration increase, may occur following a rise in insulin downregulating AQP7 expression (Kishida et al., 2001; Kondo et al., 2002), or a modification of the *Aqp7* gene affecting the expression or the activity of AQP7. Insulin resistance characterized by increase in blood glucose (due to decreased glucose uptake by tissues) and increase in insulin levels may then also prompt an increase in β-cells intracellular glycerol concentration (Virreira et al., 2011).

### Metabolic Diseases

Very little data are available in the literature concerning the expression and function of aquaglyceroporins in the endocrine pancreas in relation to metabolic diseases. One recent study, however, has shown that obese rats pancreas had increased *Aqp7* mRNA and protein levels, as compared to lean rats, with *Aqp7* transcripts being negatively correlated with total ghrelin levels and glucagon-like peptide 1 (GLP-1) (lower in obese rats) (Méndez-Giménez et al., 2017). In addition, sleeve gastrectomy in obese rats restored the altered AQP7 expression in endocrine pancreas and improved pancreatic β-cell function (Méndez-Giménez et al., 2017). Further studies will be required to determine if AQP7 could become a useful drug-target in β-cells for the treatment of metabolic diseases.

## Aquaglyceroporins as Drug Targets for Metabolic Diseases

A variety of drugs have been identified as capable of modulating either the expression and/or the activity of aquaglyceroporins. The effects as well as the potential therapeutic use of aquaglyceroporin modulators are described with main focus on their relationship to metabolic diseases. More in general, most AQPs trigger strong interest for their pharmacological potentials in medical treatment of pathologies of high epidemiological impact such as the oxidative stress-related diseases (Tamma et al., 2018).

### Gold-Based Compounds and Nucleoside Analogs

Some gold(III)-base compounds have been shown to act as modulators of aquaglyceroporins. The gold(III) compound complex [Au(phen)Cl_2_]Cl (phen = 1,10-phenanthroline; Auphen) has been shown to be a very selective and potent blocker of AQP3 by binding to its selectivity filter domain, and to the side chain of Cys40 located close to the constriction pore (Martins et al., 2012; Serna et al., 2014; de Almeida et al., 2017). Similarly, Hg^2+^, a non-selective AQPs inhibitor, has been shown to inhibit AQP3 glycerol permeability by interacting with Cys40, while inhibiting water flux due to the displacement of Arg128 concomitant to rupture of the hydrogen bond with Phe147 (Spinello et al., 2016). The Auphen complex was also shown to inhibit both mouse and human AQP7 (Madeira et al., 2014a). By homology modeling, molecular docking studies, and non-covalent docking studies, Auphen was shown to block AQP7 by direct binding to Met47, located in the pore entrance facing the cytoplasm (Madeira et al., 2014a). From a cellular point of view, Auphen was shown to inhibit cell proliferation of several cell types, including 3T3-L1 cells (Serna et al., 2014).

Other gold compounds, including [Au(bipy)Cl_2_]^+^ (bipy = 2,2′-bipyridine; Aubipy), have been shown to also inhibit AQP3 (for review see Martins et al., 2012, 2013; Beitz et al., 2015; de Almeida et al., 2017; Graziani et al., 2017; Soveral and Casini, 2017).

AQPs have been shown to contribute to cell migration, metastasis and angiogenesis (Verkman et al., 2008). In this context, via its glycerol-mediated transport, AQP3 is involved in epidermal cell proliferation where *Aqp3* knockout mice displayed both impaired epidermal cell proliferation and resistance to skin tumorigenesis (Hara-Chikuma and Verkman, 2008). Interestingly, high AQP3 expression has been associated with other types of cancers (for review, see Marlar et al., 2017), including for example colorectal carcinoma (Moon et al., 2003), pancreatic ductal carcinoma (Direito et al., 2017; Huang X. et al., 2017), hepatocellular carcinoma (Chen et al., 2016), lung cancer (Liu et al., 2007), and gastric adenocarcinoma (Huang et al., 2010; Chen et al., 2017). In addition, AQP3 plays a role in cancer cell invasion and in aggravation of epithelial-to-mesenchymal transition (for review, see Marlar et al., 2017). In addition, some nucleoside analogs used in chemotherapy of solid tumors, 5′-deoxy-5-fluorouridine (5′-DFUR) and 2′-deoxy-2′,2′-difluorocytidine (gemcitabine), stimulate AQP3 expression and cell cycle arrest (Trigueros-Motos et al., 2012). Glycerol transport, but also hydrogen peroxide transport, could mediate the downstream effect of increased AQP3 expression leading to cell cycle arrest and cytotoxicity (Miller et al., 2010).

The potential beneficial use of AQP3 inhibitors or modulators (decreasing AQP3 expression) remains to be evaluated for cancer treatment. In addition, radiolabeled glycerol could be used as a molecular probe targeting aquaglyceroporins to estimate their expression in different tissues *in vivo*, including tumors (Saito et al., 2013).

In adipose tissue, aquaglyceroporins, and in particular AQP7, represent potential drug target for the treatment of obesity and metabolic syndrome (Frühbeck et al., 2006; Maeda, 2012; Verkman, 2012). As AQP3 expression is increased in mouse 3T3-L1 differentiated into adipocytes (Chiadak et al., 2016, 2017), and in epididymal and subcutaneous adipocytes from high-fat diet rats developing obesity (Méndez-Giménez et al., 2017), and thereby could be involved in adipose tissue hyperplasia during obesity, AQP3 might also become an additional interesting drug target for the treatment of obesity.

### Metalloids

Metalloids, that are physiologically harmful to humans, are still being used in the form of arsenic or antimony containing drugs to treat certain diseases and forms of cancers (Mukhopadhyay and Beitz, 2010). *Xenopus laevis* oocytes injected with aquaglyceroporins cRNA revealed that human AQP3, AQP7, and AQP9 conducted the movement of AsIII across cell, while AQP10 did not (Liu et al., 2002). However, in contrast to these observations, human *Aqp10* knockdown in human colorectal cell line Caco-2 resulted in reduced As^III^ accumulation, suggesting that AQP10 might facilitate As^III^ transcellular flow (Calatayud et al., 2012) as well. Whether human AQP10 is permeable to As^III^ awaits additional studies. Mouse and rat AQP7 and AQP9 were also shown to be permeable to As^III^ (Liu et al., 2002; Carbrey et al., 2009). Monomethylarsonous acid (MMA^III^), one of the major methylation products of inorganic arsenic, can permeate through rat AQP9, human AQP9 and mouse AQP7, but not human AQP7 (Liu et al., 2006; Liu, 2010; McDermott et al., 2010). In addition, human AQP9 is permeable to two other major methylation products of inorganic arsenic: monomethylarsonic acid (MMA^V^) and dimethylarsenic acid (DMA^V^) (McDermott et al., 2010). Human AQP9 was also found to be permeable to lactate, ionic selenite and monomethylselenic acid (MSeA) (Geng et al., 2017). MSeA could be used for the prevention and treatment of several cancer types, such as pancreatic cancer (Wang et al., 2014) and lung cancer (Swede et al., 2003), as it induces cell cytotoxicity. Lower AQP9 expression has been related with non-response to adjuvant chemotherapy, comprising amongst others a nucleoside analog, in colorectal cancer (Dou et al., 2013). In addition, AQP9 was recently shown to enhance the cytotoxic effect of nucleoside analogs in colorectal cancer, and could thereby be used as a novel predictor for the benefit of nucleoside analog chemotherapy in the disease (Huang D. et al., 2017). In hepatocellular carcinoma decreased AQP9 expression has been associated with apoptosis resistance, invasion and epithelial-to-mesenchymal transition (Jablonski et al., 2007; Padma et al., 2009; Li et al., 2016; Chen et al., 2016). Downregulation of aquaglyceroporin expression may lead to a metalloid resistant and/or adjuvant resistant phenotype. However, drugs aiming at specifically increasing AQP9 expression in tumor cells will sensitize them to metalloid compounds and favor the anticipated cytotoxic effects (Mukhopadhyay et al., 2014). These data emphases the efficacy of using combined therapeutic approaches acting in synergy for the treatment of cancers. Consequently, chemotherapy would benefit from the identification of novel drugs that could specifically increase aquaglyceroporin expression in cancer cells.

Due to its permeability to glucogenic glycerol (Jelen et al., 2011; Calamita et al., 2012; Geng et al., 2017), AQP9 represents an interesting drug target in the pharmacological modulation of gluconeogenesis.

### Antidiabetic Drugs

Troglitazone (a thiazolidinedione activating PPARγ) and tolbutamide (a sulfonylurea blocking potassium channels) were shown to significantly decrease AQP3 expression in Caco-2 cells, while metformin [belonging to the beguanide family and activating AMP-activated protein kinase (AMPK)] and tolbutamide did not alter AQP3 expression (Asai et al., 2006). PPARγ agonists have also been shown to increase adipose tissue AQP7 expression (Kishida et al., 2001; Kondo et al., 2002; Lee et al., 2005). It remains to be determined if rosiglitazone (another thiazolidinedione activating PPARγ) and metformin, that restored insulin secretion in pancreatic β-cells chronically exposed to free fatty acids or high glucose (Patanè et al., 2000; Richardson et al., 2006), increased AQP7 expression. Further studies are necessary to study the effects of antidiabetic drugs on aquaglyceroporin expression, pertaining to key tissues involved in the control of energy metabolism, including adipose tissue, liver and endocrine pancreas. As a result, this would allow a better understanding of the mechanisms accountable for the beneficial effects of antidiabetic drugs in metabolic diseases, and develop new therapeutic strategies. Therapeutic synergy between metformin, suppressing hepatic gluconeogenesis (Petersen et al., 2017) and restoring insulin secretion (Patanè et al., 2000; Richardson et al., 2006), and drugs, specifically decreasing aquaglyceroporin hepatic expression or activity, could be beneficial for the treatment of T2D characterized by increased hepatic gluconeogenesis and hepatic lipid accumulation leading to hepatic insulin resistance (Petersen et al., 2017).

### Other Compounds

Phloretin, a natural antioxidant phenol acting as a potent inhibitor of glycerol fluxes (Abrami et al., 1996), inhibits urea and glycerol permeability in rat and human hepatocyte cell membranes (Calamita et al., 2012; Jelen et al., 2012; Rodríguez et al., 2014). Recently, it has been shown that intraperitoneal administration of 10 mg/kg of phloretin (twice weekly) in mice fed a high-fat diet during 12 weeks reverted obesity, prevents weight gain, hyperinsulinemia, glucose intolerance, insulin resistance, and fatty liver (Alsanea et al., 2017). Moreover, intraperitoneal administration of 10 mg/kg of phloretin (twice weekly) in mice made obese by submitting them for 6 weeks to a high-fat diet improved insulin levels, insulin resistance and hepatic liver accumulation, but did not modify body weight or fasting blood glucose levels (Alsanea et al., 2017). These data suggest that phloretin could represent an additional pharmacological tool for the treatment of obesity and its associated metabolic diseases.

Obese *Aqp9* knockout mice are characterized by reduced fasting blood glucose levels as compared to obese WT *Aqp9* mice (Rojek et al., 2007). Several AQP9 small molecule inhibitors were identified by screening a commercial compound library using a cell-based permeability assay (Jelen et al., 2011; for review see Beitz et al., 2015). The compounds showing the highest efficiency in blocking AQP9 were subjected to *in silico* molecular docking to a human AQP9 model and the compounds showing good docking properties were functionally tested using Chinese Hamster Ovary (CHO) cells expressing AQP9 (Jelen et al., 2011). The putative binding sites of the most interesting compounds were then evaluated by site-directed mutagenesis and a cell water permeability assay of mutated AQP9 channels (Wacker et al., 2013). Few additional patent compounds have been claimed to modulate AQP9 expression or activity, thereby being of therapeutic interest for the treatment of diseases (for review see Soveral and Casini, 2017). In humans, AQP9 is expressed almost exclusively in the liver parenchyma with evident pharmacological advantages as a drug-target pathway. Given the relevance of AQP9 in facilitating the entry of glycerol, the carbon backbone of TAG, into hepatocytes, it is reasonable to think that small chemical compounds with micromolar-submicromolar potency in blocking selectively the AQP9 channel, may be effective in counteracting the aberrant hepatic fat accumulation underlying NAFDL/NASH, pathologies for which several potential therapeutic approaches have been proposed while no established therapy does exist (for a review see Calamita and Portincasa, 2007). As for T2D, structural analogs of HTS13286 featuring pharmacological and clinical sustainability offer worthy potential as new options in preventing end-stage liver disease, from simple steatosis to NAFLD/NASH and consequent liver fibrosis and hepatocarcinoma. Phytochemical modulation of AQP9 expression by nutraceutics counteracting excess hepatic lipid accumulation (Cataldo et al., 2017; Tesse et al., 2018) is also worthy of investigation in preventing or improving prognosis of NAFLD/NASH. The heterocyclic compound HTS13286 is a selective and potent blocker of AQP9 with an IC_50_ of 1.5 μM (Jelen et al., 2011), however, its scarce water solubility hampers its use *in vivo*. Design and synthesis of structural analogs of HTS13286 with improved hydrophilicity, pharmacodynamic and pharmacokinetic properties is therefore a worthwhile task.

## Conclusion and Future Perspectives

A body of evidence indicates involvement of Adipose liver and endocrine pancreas aquaglyceroporins in the metabolic and energy control function exerted by these tissues. Metabolic relevance is also testified by the alterations in expression levels and regulation to which they undergo in clinical disorders associated with metabolic and energy dysfunction. While a number of reports have described aquaglyceroporins modulation by phytochemical compounds preventing or improving chronic metabolic diseases, investigation of their pharmacological potential is just picking up speed. New and more sustainable aquaglyceroporin inhibitors have been identified while others are subject to ongoing research. Development of therapeutic strategies targeting aquaglyceroporins may therefore offer promise for the management of a large spectrum of clinical disorders including metabolic and energy balance diseases. Overall, the study of biologically active phytochemicals and synthetic compounds as modulators of aquaglyceroporin expression or function is an emerging topic in which new and important achievements are anticipated.

## Author Contributions

CD wrote the first draft of the manuscript. GC, JP, and CD wrote sections of the manuscript. All authors contributed to manuscript revision, read and approved the submitted manuscript.

## Conflict of Interest Statement

The authors declare that the research was conducted in the absence of any commercial or financial relationships that could be construed as a potential conflict of interest.
